# Identification and validation of reference genes for qPCR in the terrestrial gastropod *Cepaea nemoralis*

**DOI:** 10.1371/journal.pone.0201396

**Published:** 2018-08-29

**Authors:** Susanne Affenzeller, Nicolas Cerveau, Daniel John Jackson

**Affiliations:** Department of Geobiology, Georg-August University of Göttingen, Göttingen, Germany; Nazarbayev University, KAZAKHSTAN

## Abstract

Identifying and understanding mechanisms that generate phenotypic diversity is a fundamental goal of evolutionary biology. With a diversity of pigmented shell morphotypes governed by Mendelian patterns of inheritance, the common grove snail *Cepaea nemoralis* (Linnaeus, 1758) has been a model for evolutionary biologists and population geneticists for decades. However, the genetic mechanisms by which *C*. *nemoralis* generates this pigmented shell diversity remain unknown. An important first step in investigating this pigmentation pattern is to establish a set of validated reference genes for differential gene expression assays. Here we have evaluated eleven candidate genes for reverse transcription quantitative polymerase chain reaction (qPCR) in *C*. *nemoralis*. Five of these were housekeeping genes traditionally employed as qPCR reference genes in other species, while six alternative genes were selected *de novo* from *C*. *nemoralis* transcriptome data based on the stability of their expression levels. We tested all eleven candidates for expression stability in four sub-adult tissues of *C*. *nemoralis*: pigmented mantle, unpigmented mantle, head and foot. We find that two commonly employed housekeeping genes (*alpha tubulin*, *glyceraldehyde 3-phosphate dehydrogenase*) are unsuitable for use as qPCR reference genes in *C*. *nemoralis*. The traditional housekeeping gene *UBIquitin* on the other hand performed very well. Additionally, an *RNA-directed DNA polymerase* (*RNAP*), a *Potassium Channel Protein* (*KCHP*) and a *Prenylated Rab acceptor protein 1* (*PRAP*), identified *de novo* from transcriptomic data, were the most stably expressed genes in different tissue combinations. We also tested expression stability over two seasons and found that, although other genes are more stable within a single season, *beta actin* (*BACT*) and *elongation factor 1 alpha* (*EF1α*) were the most reliable reference genes across seasons.

## Introduction

**Fig 1:**
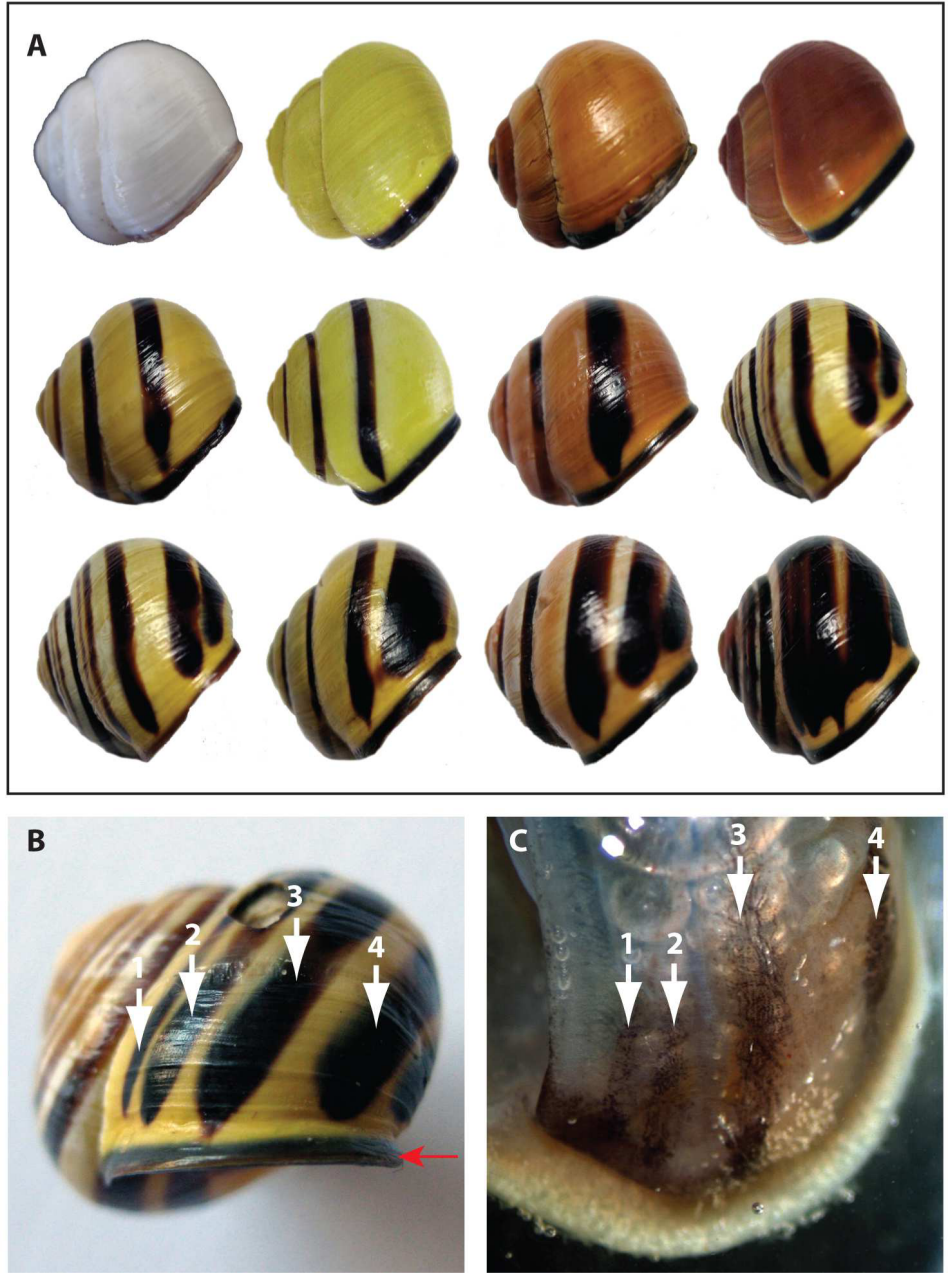
Pigmentation and banding patterns display marked variation in *C*. *nemoralis*. **A.** Representative shell colour morphs of *C*. *nemoralis*. Shell background colour can range from white to yellow and pink to dark orange or brown. Banding patterns include zero to five bands that can merge or be distinct. **B.** A shell with a yellow background and a typical banding pattern. The lateral pigmented band at the leading edge of the shell that indicates a fully differentiated snail is indicated with a horizontal red arrow. **C.** Banding patterns in the shell are reflected in the pigmentation of the underlying mantle tissue, and facilitate the isolation of populations of cells for separate RNA extractions and gene expression profiling. The mantle tissue presented here was responsible for both constructing and pigmenting the shell shown in B. The individual pigmented bands in the mantle are numbered and correspond to those indicated on the shell.

Variation in the pigmentation and patterning of the shell of the common grove snail *Cepaea nemoralis* (Linnaeus, 1758), has been studied by ecologists and evolutionary biologists for decades [1, 2, 3]. Shell background colour can range from a very light yellow to dark pink, orange and brown with up to five dark brown bands. The shell itself and any patterns of pigmentation are produced by the underlying mantle tissue (Fig 1).

Early studies of *C*. *nemoralis* focused on the distribution of colour morphs and attempted to identify the evolutionary pressures that may have generated this variation. Hypotheses such as crypsis and body temperature control have been investigated [4, 5, 6, 7, 8], however no single mechanism can yet convincingly account for the observed distributions of variably pigmented populations of *C*. *nemoralis*. Nonetheless the variable banding patterns and background colours make it an excellent system to study the underlying molecular mechanisms of complex pigmentation patterns [9, 10, 11]. The shell polymorphism present in *C*. *nemoralis* is thought to be controlled by a supergene, a region of genomic DNA containing tightly linked and neighbouring loci controlling different aspects of the observed variation, such as shell background colour and the number of dark pigmented bands [12, 13]. A variety of next generation sequencing (NGS) and proteomic approaches have been applied to identify this supergene in recent years [13, 14, 15]. However the precise genomic loci and mechanism responsible for these variable pigmented phenotypes remain unknown.

Partly hampering these efforts is a lack of established methods in *C*. *nemoralis* to independently verify candidate pigmentation genes. Reverse transcription quantitative polymerase chain reaction (qPCR) is a commonly used molecular technique to measure gene expression levels between biological samples. When employing qPCR to measure gene expression levels between samples it is critical to normalise values using reference genes that must be stably expressed across the conditions being investigated [16]. Concerningly, previous studies have shown that commonly employed reference genes are often far from stably expressed [17, 18, 19]. It is therefore important to validate any panel of reference genes intended for qPCR prior to conducting analyses of gene expression.

To date, only a handful of molluscan species have had reference genes tested for stable expression [20, 21, 22, 23, 24], and in *C*. *nemoralis* this has never been performed. Our aim was therefore to validate a set of reference genes for qPCR from a selection of traditionally used reference genes and alternative genes identified *de novo* from transcriptome data. These genes could then be used for future shell pigmentation and patterning studies in this organism.

## Material and methods

### Transcriptome sequencing

Three sub-adult individuals of *C*. *nemoralis* (i.e. individuals actively laying down shell material without the terminal pigmented lateral band present in all mature individuals shown in Fig 1) were collected at the University of Göttingen, Germany (51°33'24.0"N 9°57'27.3"E). These three individuals comprise three biological replicates. Two RNA extractions were performed for each individual from the most distal edge of the mantle; one from a dark pigmented region and one from an adjacent unpigmented region (see Fig 1C). Total RNA was extracted using Trizol (Molecular Research Center, #TR118) according to the manufacturer’s instructions. Library preparation and sequencing was carried out at the Transcriptome and Genome Analysis Laboratory, University Medical Center Göttingen, Germany. A TrueSeq RNA Sample Kit (Illumina, Cat. N°RS-122-2002) was used to generate an mRNA library using 500 ng of total RNA. During TrueSeq Illumina library construction rRNA were depleted with oligo dT beads. Quantitation of cDNA libraries was performed using the QuantiFluorTM dsDNA System (Promega) and the size range of the final cDNA libraries was determined using a Bioanalyzer 2100 from Agilent (280 bp). Stranded sequencing was conducted on the Illumina HiSeq 2000 platform to generate 100 bp paired-end reads. More than 470 million paired reads were generated. This raw data is the basis of our ongoing efforts to identify genes involved in shell pigmentation in *C*. *nemoralis* and will be reported in full elsewhere, however all sequence data relevant to the analyses performed here have been made publicly available (see below). Trimmomatic [25] was used to remove low quality reads and adapter sequences. Reads from the six samples were pooled and treated together for the assembly process. Pooled reads were assembled *de novo* using our assembly pipeline [26]. Briefly, we employed three assembly packages with unique assembly strategies: Trinity V2.0.3 [27], CLC Genomics Workbench (www.qiagenbioinformatics.com) and IDBA-tran V1.1.1 [28]. The CLC assembler was run using a word size of 20 base pairs (bp), a bubble size of 50 bp, with reads mapped back to the transcriptome using default parameters. IDBA_tran was run with kmer values ranging from 20 to 100 bp with a step size of 10 bp. Trinity assemblies were run with default parameters and a k-mer value of 25. Contigs with coding regions larger than 100 amino acid residues were merged into a concatenated assembly, and redundancy was removed as described [26].

Differential gene expression analysis was performed using CLC Genomics Workbench (www.qiagenbioinformatics.com). A matched pairs experimental design (1 matched pair from each individual replicate) was established to compare pigmented mantle and unpigmented mantle samples. Trimmed paired-end reads from each condition were mapped to the reference assembly using total gene read counts.

### Selection of reference gene candidates

Candidate reference genes were selected from our transcriptome data in two fundamentally different ways. First, a set of five commonly employed housekeeping genes were selected from the literature and subsequently identified in our transcriptome data. These included *Alpha Tubulin* (*ATUB;* Accession MH035488), *Beta Actin* (*BACT;* Accession MH035489), *Elongation Factor 1 alpha* (*EF1α;* Accession MH035491), *Glyceraldehyde 3-phosphate dehydrogenase* (*GAPDH;* Accession MH035493), *UBIquitin* (*UBI;* Accession MH035498). Second, six candidates were chosen based on the low standard deviation in their total read counts across all six transcriptomes. These six alternative candidates were annotated using SmartBLAST against SwissProt, and tBLASTx against ref-seq. These candidates were identified as *DNA Repair Protein* (*DNARP;* Accession MH035490), *Fibronectin type III domain containing protein* (*FIB3;* Accession MH035492), *GTP-binding protein* (*GTPB;* Accession MH035494), *Potassium Channel Protein* (*KCHP;* Accession MH035495), *Prenylated Rab acceptor protein 1* (*PRAP;* Accession MH035496) and *RNA-directed DNA polymerase* (*RNAP;* Accession MH035497). Primers to all of these genes were designed using Primer3 [29, 30]. Table 1 lists all primer sequences and their resulting amplicon sizes. Primer specificity was verified with melt curve analysis and agarose gel electrophoresis.

**Table 1:** Primer sequences and amplicon sizes for all used reference genes.

Gene	Forward primer (5' to 3')	Reverse primer (5' to 3')	Amplicon size (bp)
*ATUB*	ATGGTATTCAGCCTGACGGT	AACATGTTTGCCAGCTCCTG	104
*BACT*	CAGAAGCAATGTTCCAGCCA	TGAGCCACCAGACAAGACAA	137
*DNARP*	GACCACAAGAGGACAGCTCT	GTTATCCTGCAGCACACCAC	105
*EF1α*	GTACCGGAGAGTTTGAGGCT	GAGTAAGGTGGAGTGGTGCT	133
*FIB3*	TGGATGACATCGGGGAACAA	ATCACTATCGCAACAACGGC	170
*GAPDH*	CATGTTTGTGCTGGGTGTCA	TCAGGCCCTCAACAATTCCA	137
*GTPB*	ATTCAAGCACCCGGAGAGAT	TAAGTGCCAGAACAGACGGT	107
*KCHP*	GTTGTTGCCTCAATCGTGGT	AAGCAGTCTCCAAGTCCCTC	138
*PRAP*	ATCTTGACATCTCCGCTGCT	GATTTCACGACCCATCACGG	111
*RNAP*	CGCGTGACAATGGACAAAGA	CGCCTCTCTATCCGTATCCC	102
*UBI*	AGAATGCCCCAACAAATGCT	AGAATCAGCCTCTTCTCCGG	121

### RNA extraction and cDNA synthesis

**Fig 2:**
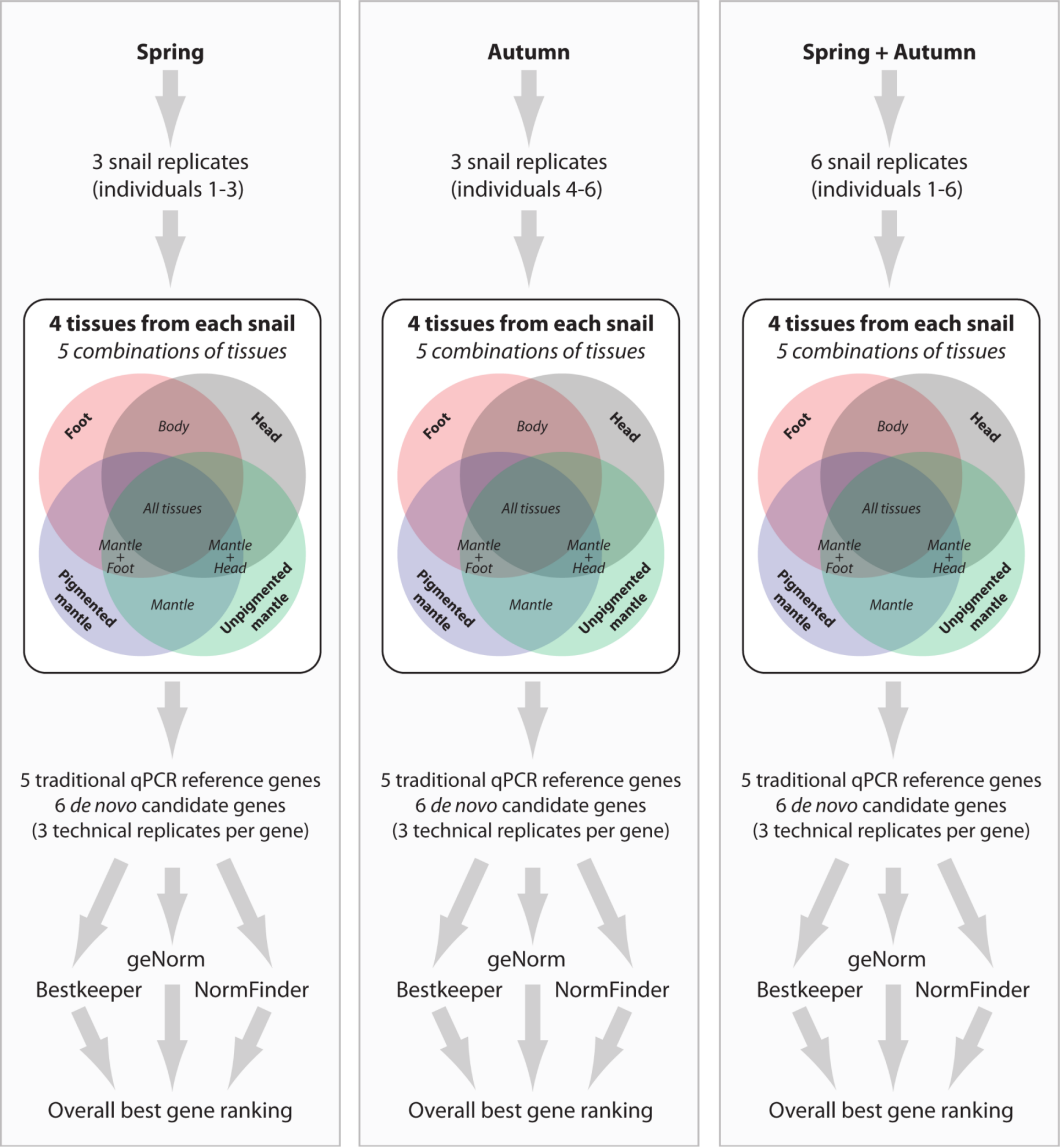
A schematic representation of our sampling and experimental design and data analysis. RNA was extracted from four different tissues derived from six snail replicates across two seasons. The four independent tissues (and five combinations thereof) and three algorithms were used to rank gene performance, and these results were then combined to find the overall best ranked genes.

Total RNA was extracted from six sub-adult individuals of *C*. *nemoralis* (independent of those used for transcriptome sequencing) collected at the University of Göttingen (51°33'24.0"N 9°57'27.3"E). Three individuals were collected in spring (May, 2017), while three were collected in autumn (September, 2017). These seasons encompass periods of relatively rapid and depressed shell growth respectively [31]. All individuals possessed a yellow background shell colour with two or more bands (Fig 1). Tissue from pigmented mantle (PM) and unpigmented mantle (UPM), foot (F) and head (H) were carefully dissected and used for a total of 24 RNA extractions using Qiazol (Qiagen, #79306) according to the manufacturer’s instructions (Fig 2). Following successful RNA extraction a DNase treatment (RQ1 RNase-free DNase, Promega, #M6101) was carried out according to the manufacturer’s instructions. Briefly, 1 μL DNase and 1 μL buffer solution were used on 8 μL of extracted RNA. The reaction was stopped with 1 μL of Promega STOP solution. The quality and integrity of RNA was verified with a Nanodrop and agarose gel electrophoresis, and a standard PCR against the *CO1* barcoding fragment was carried out to verify that the DNase treatment was effective. For each sample, 1 μg of total extracted RNA was used in a cDNA synthesis reaction containing 10 μL oligo dT (10μM), 2 μL M-MLV reverse transcriptase (RT) H–(Promega #M3682), 10 μL 5x M-MLV RT buffer (Promega), 2 μL dNTPs (10mM) and x μL nuclease free water to a total volume of 50 μL. RNA, oligo dTs and nuclease free water to a volume of 15 μL were heated to 70°C for 5 min, then cooled to 42°C. The remaining reaction components were added at 42°C and the reaction was incubated for a further 75 min. The RT was inactivated at 70°C for 15 min and the cDNA was stored at -20°C until further use.

### qPCR

All qPCR runs followed a maximum sample layout for each gene (all tissue samples included in one run) and comply with the MIQE guidelines [32]. Each run also included no template controls (NTC) for each primer pair and three inter run calibrators (IRC). *EF1α*, *RNAP* and *UBI* were randomly chosen as IRCs and were run on mixed tissue cDNA. Samples were run in triplicate, NTC and IRCs were run in duplicate. qPCR reactions contained 5 μL 2x Rotor-Gene SYBR Green PCR Master Mix (Qiagen, #204074), 0.4 μL cDNA, 0.1 μL of each primer (1μM final concentration) and 4.4 μL ddH_2_O to a final volume of 10 μL. Amplification and detection were performed on a Rotor-Gene Q (Qiagen) running Rotor-Gene Q software (version 2.0.2) with the following temperature profile: 5 min initial activation and denaturation at 95°C; 40 cycles of 5 sec denaturation at 95°C, 10 sec annealing and extension at 60°C (data collection at this step); a final melt curve analysis from 60°C to 95°C at a rate of 5 sec/1°C. PCR amplicons were also assessed on agarose gels to qualitatively visualise amplification efficiency (S1 Fig).

### Data analyses

Raw fluorescence data was exported from the Rotor-Gene software for further analysis. Baseline correction and amplification efficiency correction were performed using LinRegPCR [33]. Inter run correction was performed using Factor-qPCR [34]. The resulting corrected cycle threshold (Cq) values were used to calculate the geometric means of technical replicates. The expression stability of each candidate reference gene was calculated for each of the six individuals independently, before being averaged across individuals. We also analysed each candidate reference gene in all four tissues independently (Pigmented Mantle (PM), Unpigmented Mantle (UPM), Foot (F) and Head (H), and also after pooling all four tissues (All). Finally we tested different combinations of tissues: Body (F + H), Mantle (PM + UPM), Mantle and Foot (PM + UPM + F) and Mantle and Head (PM + UPM + H). These sample combinations were also run for spring animals, autumn animals and a season independent ‘total’ (spring + autumn) set (Fig 2). This allowed us to identify the most appropriate reference genes for our research interests (shell pigmentation), and to also assess the broader stability of these genes across diverse tissue types and seasons. We tested gene expression stability using three different algorithms: BestKeeper (which calculates efficiency corrected descriptive statistics) [35], NormFinder (which is based on inter- and intra-group variation calculations for reference gene stability testing) [36] and geNorm (which estimates stability of reference genes based on pairwise comparison of gene specific variation) [37]. Data was transformed with the following formula for use in NormFinder and geNorm:

$$\text{Transformed data}={\text{sample efficiency}}^{(\text{minCq}\left(\text{Gene}\right)–\text{Cq}\left(\text{Sample}\right)}$$

Where sample efficiency is the amplification efficiency of the reaction, minCq(Gene) is the lowest observed cycle threshold per gene, and Cq(Sample) is the observed cycle threshold for the sample [35]. To summarise the results of all three algorithms, each candidate reference gene was ranked from the most (1) to the least (11) stably expressed and then averaged across algorithm (see the Supplementary Material for the results of each individual algorithm). Data preparation for BestKeeper, NormFinder and geNorm, as well as ranking, and descriptive statistics and visualisation of results were carried out in Excel 2016 MSO (v16.0.8326.2096, Microsoft).

## Results

### Variation between individual snails influences the ranking of candidate reference genes

**Fig 3:**
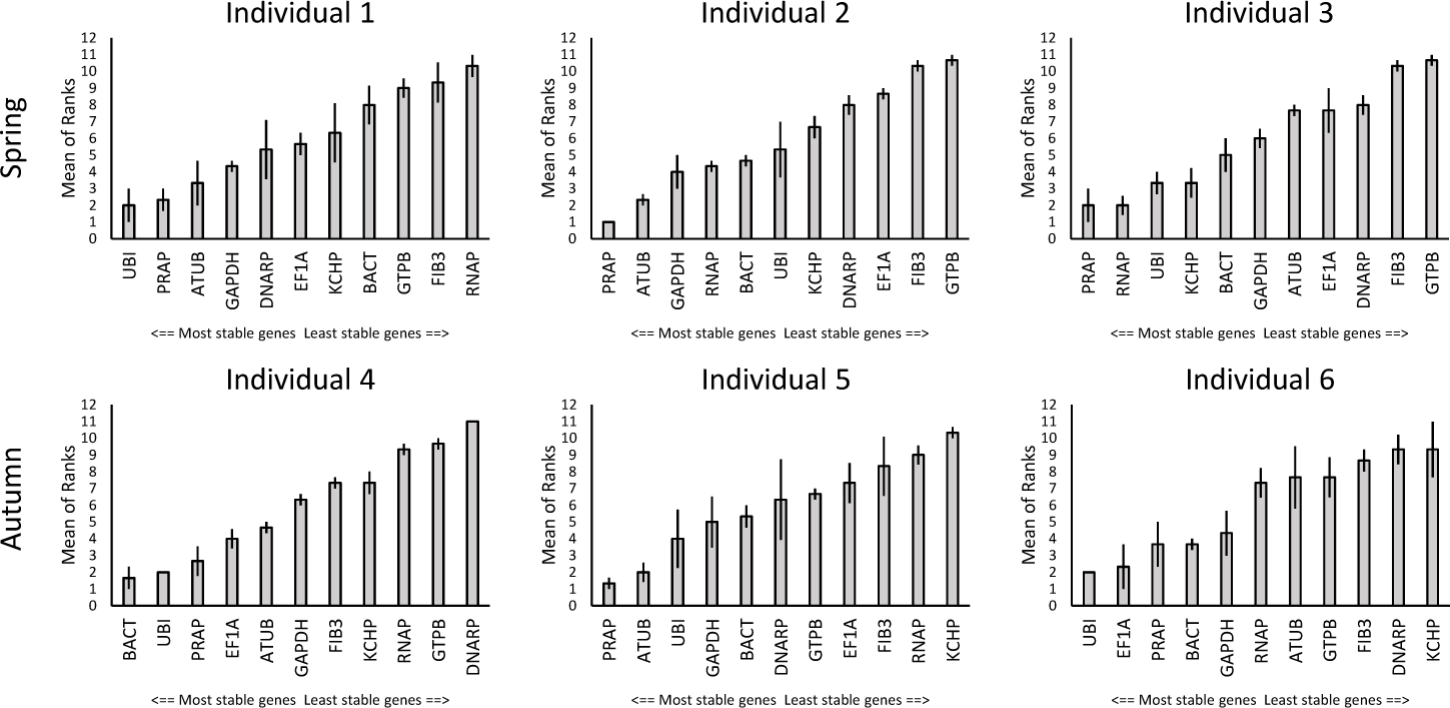
Expression stability ranking across all tissues within each individual snail. Means of ranked data generated by three algorithms (BestKeeper, geNorm and NormFinder). Error bars indicate standard error.

The ranking stability of candidate reference genes on the pooled tissue data from each individual snail revealed some differences. However the overall mean of ranks across all three algorithms revealed *PRAP* to be among the top three most stably expressed genes in all individuals. Only one other gene, *UBI* performed just as well, except in individual 2. Additionally *ATUB*, *GAPDH*, *RNAP*, *BACT* and *EF1α* showed good overall ranks in single individuals (Fig 3).

### Tissue type and season influence the ranking of candidate reference genes

Within Foot tissue *KCHP*, *UBI* and *PRAP* were identified as the best candidate reference genes for the spring subset (individuals 1–3). For animals sampled in autumn (individuals 4–6) *BACT*, *GTPB* and *ATUB* performed the best in Foot tissue. In combining all Foot tissue seasons (spring + autumn) *BACT*, *EF1α* and *UBI* were identified as the best reference genes.

In Head tissue *BACT*, *RNAP* and *DNARP* showed the best performance in spring samples, while *BACT*, *PRAP* and *GAPDH* performed best in autumn samples. In the spring + autumn Head tissue set *BACT* and *EF1α* were revealed as the best genes.

*GAPDH* and *UBI* performed best in Pigmented Mantle tissue in spring, whereas *GAPDH* and *GTPB* outperformed all other genes in autumn. In the spring + autumn Pigmented Mantle sample set *BACT* and *EF1α* performed best.

**Fig 4:**
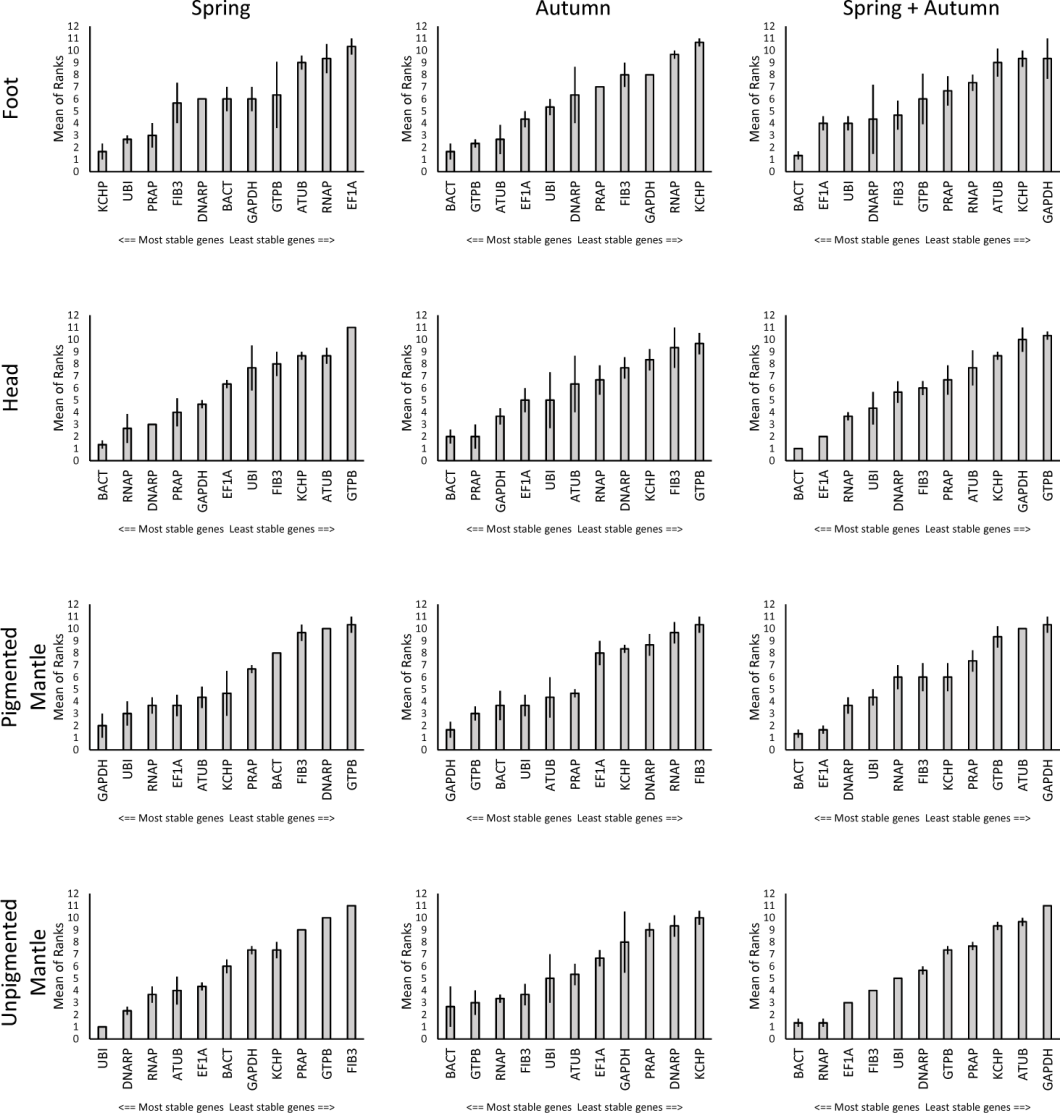
Expression stability ranking within each tissue type. Means of ranked data generated by three algorithms (BestKeeper, geNorm and NormFinder). Error bars indicate standard error.

In Unpigmented Mantle tissue extracted in spring *UBI* and *DNARP* showed the best results, while in the autumn subset *BACT* and *GTPB* performed best. Combining both seasonal subsets of Unpigmented Mantle identified *BACT* and *RNAP* as the best reference genes in all three algorithms (Fig 4).

### Tissue type combinations influence the ranking of candidate reference genes

**Fig 5:**
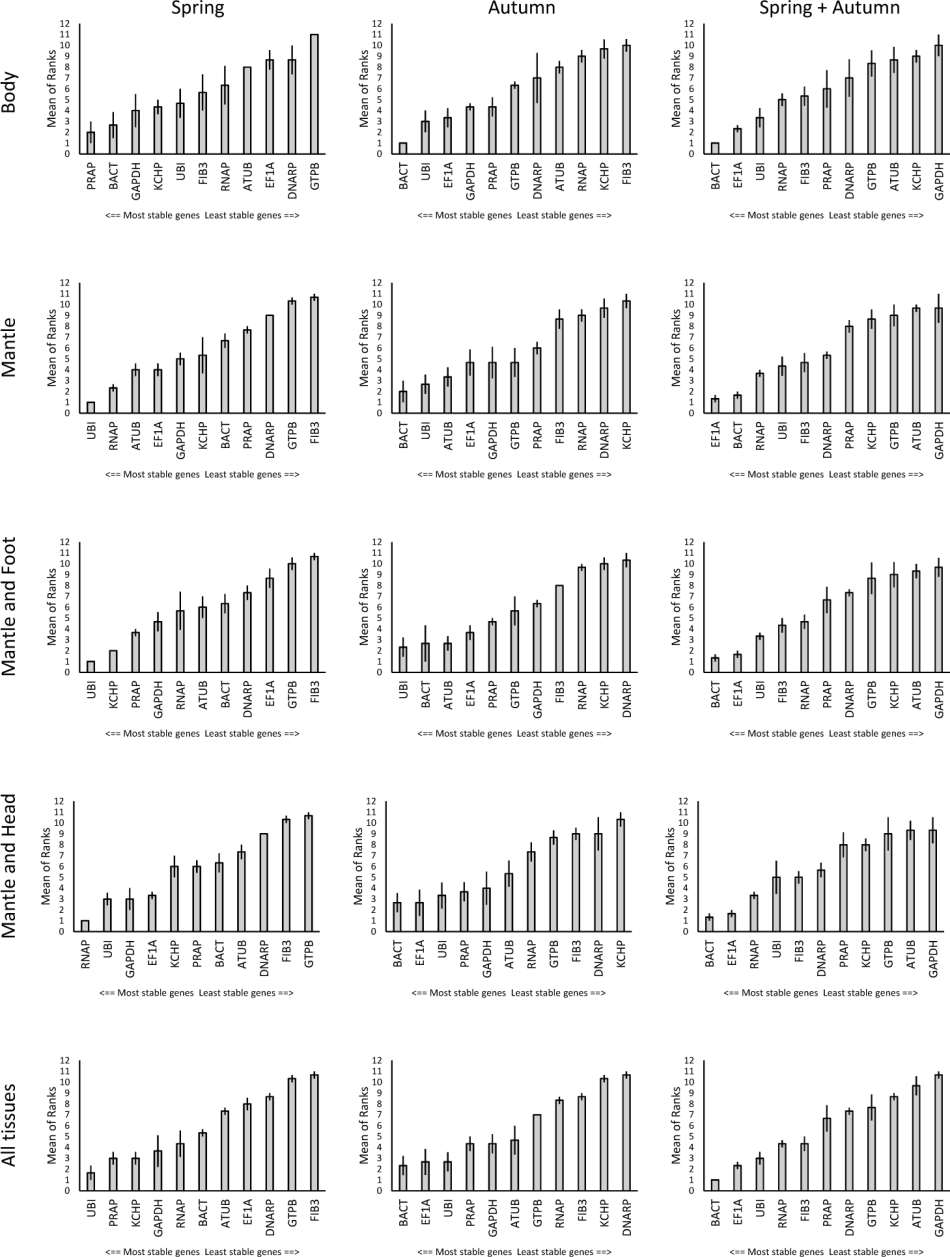
Expression stability ranking of combined tissue types. Body (Foot and Head); Mantle (Mantle pigmented and unpigmented); M+F (Mantle and Foot); M+H (Mantle and Head); All (all tissue sets). Means of ranked data generated by three algorithms (BestKeeper, geNorm and NormFinder). Error bars indicate standard error.

When we combined more than one tissue type and repeated our analyses *UBI* emerged as the most stably expressed gene for all three algorithms in spring Mantle (PM + UPM), spring and autumn Mantle and Foot (PM + UPM + F) and spring All (PM + UPM + F + H). Considering the spring subset of individuals *RNAP* was revealed as a second candidate gene in Mantle, Mantle and Foot, as well as in All. *KCHP* performed second best with *PRAP* performing equally well in All. For Body tissue (F + H) *PRAP* and *BACT* performed best overall. *RNAP*, *UBI* and *GAPDH* tested as best candidate genes in the data set Mantle and Head (PM + UPM + H). In autumn animals *BACT* was revealed as the best overall candidate in all remaining multiple tissue combinations (all except M + F), followed by *EF1α* in All and in M + H, and *UBI* in Body and Mantle tissue sets. In the combined analyses of all extractions *BACT* and *EF1α* performed best in all multiple tissue sets across the three algorithms (Fig 5).

Results for all three algorithms over all data sets, as well as the mean of ranks over all three algorithms results are summarized in S1 Table. The results generated by each of the three individual algorithms on all datasets are presented and discussed in the Supplementary Material (S2–S7 Figs).

## Discussion

*C*. *nemoralis* holds great promise as a model for investigating the molecular mechanisms that regulate gastropod shell pigmentation. As a first step towards this goal we have assayed a total of eleven candidate reference genes for their suitability in qPCR experiments. Five of these were genes commonly employed in other model systems as reference genes, and six were alternative genes we identified *de novo* as having potentially stable expression profiles across pigmented and unpigmented mantle tissue. In our analyses one of the major challenges to obtaining robust measures of gene expression was highlighted; expression levels of specific genes can vary significantly between individuals, across tissue types and across seasons. One prominent example is our single tissue analyses of *GAPDH*. Although this gene is stably expressed within spring and autumn in Pigmented Mantle, it is ranked as the least stable gene across seasons (Fig 4). This is because *GAPDH* seems to have a very uniform expression within a given season, but expression levels change drastically between seasons. This highlights the importance of biological replicates, experimental design and reference gene choice to any qPCR experiment when samples across tissue types and time points are to be compared.

Despite this variability some patterns emerged, perhaps most importantly that *PRAP* consistently tested within the top three candidates for all six individuals across seasons, tissue type and algorithm (Fig 3). The often employed house keeping genes *BACT*, *UBI* and *EF1α* tested well in different sub sets of tissues and tissue combinations. The traditionally employed house keeping genes *ATUB* and *GAPDH* however did not perform consistently. Although this is the first time the genes *PRAP*, *RNAP* and *KCHP* have been considered as potential reference genes for qPCR, they performed very well in specific data sets and across all algorithms, and we propose they should be considered in all qPCR studies in *C*. *nemoralis*. We suggest they may also be applicable for qPCR in other gastropod species, and perhaps even more widely, and should therefore be included in reference gene testing.

Because the literature concerning the identification of stable reference genes in molluscs is extremely small we can compare our results to these previous efforts. In a study testing eight reference genes in the pond snail (*Bellamya aeruginosa*) the highly expressed *ribosomal protein L7* gene was identified as the most stably expressed [24]. However several studies in diverse animals have demonstrated that ribosomal genes are not reliable candidates for stable expression [20, 21, 38], therefore we did not consider them for investigation here. *BACT* performed well in our study of *C*. *nemoralis*, both in single and multiple tissue sets, as well as in both seasons separately. It is also one of the genes that displayed stable expression across seasons. However it did not behave ideally in all data sets, a result that agrees with differences in *BACT* expression between tissues and treatments in other molluscs [20, 21, 22, 23, 24]. Our results also identified the traditional housekeeping gene *UBI* as a good candidate reference gene in multiple data sets in both spring, autumn and spring + autumn (e.g. spring All tissues and Mantle and Foot, autumn Body and Mantle, spring + autumn All tissues, Body and Mantle and Foot). This agrees with results reported in larval and adult cephalopods [20, 23]. In *Mytilus edulis* and *Haliotis discus EF1α* could be identified as a stable reference gene [21, 22]. Our data agrees with this when considering comparisons across seasons and in some autumn tissue sets. However our results for tissue samples taken in spring did not support *EF1α* as a good candidate, agreeing with the reported unstable expression of this gene across different adult tissues in *O*. *vulgaris* [20]. *GAPDH* performed well in Pigmented Mantle, but showed variable expression in other tissue combinations and especially across seasons, an instability already observed in *Haliotis* [21]. Another frequently employed housekeeping gene *ATUB* displayed very tissue dependent results in our analyses and did not in general rank well under many conditions. We therefore cannot recommended *GAPDH* or *ATUB* as references genes for qPCR in *C*. *nemoralis*.

To summarise, we recommend different sets of reference genes for qPCR studies in *C*. *nemoralis* depending on the research question. By mining digital gene expression profiles we identified *PRAP*, *RNAP* and *KCHP de novo* as candidate reference genes for qPCR that performed well under a range of conditions. For our particular research interests in shell pigmentation *EF1α* and *BACT* across seasons, *BACT* and *UBI* in autumn and *UBI* and *RNAP* in spring appear to be the most appropriate reference genes. Three of these genes (*UBI*, *EF1α* and *BACT*) can also be used for studies including more diverse tissue samples of *C*. *nemoralis*. Our results clearly demonstrate that when choosing reference genes for qPCR, the selection must not only be adapted to the target species, but also to the intended experimental design and should therefore consider variables such as seasonal variation, tissue specificity, environmental factors and the method of analysis.

## Supporting information

S1 Table Expression stability indices of reference genes tested in all data sets. Results for Individuals 1 to 6 (Ind1-6), Foot (F), Head (H), Pigmented Mantle (PM), Unpigmented Mantle (UPM), Body (F + H), Mantle (PM + UPM), Mantle and Foot (PM + UPM + F), Mantle and Head (PM + UPM + H) and All tissues (PM + UPM + F + H) spring, autumn and all year data sets for Mean of Ranks, BestKeeper, geNorm and NormFinder. Mean of Ranks values represent the ranked means of all three algorithms, where 1 is the most stable gene and 11 is the least stable gene. geNorm values represent M values as calculated by the algorithm. NormFinder values are Stability values as calculated by the algorithm. BestKeeper values represent Standard Deviation of Cq as calculated by the algorithm.
(XLSX)

S1 Fig Qualification of qPCR amplification specificity by gel electrophoresis. Representative images of agarose gel electrophoresis after qPCR amplification for each of the tested reference genes. Size ladders on the left indicate fragments from 100 to 1000 base pairs.
(TIF)

S2 Fig qPCR results of reference gene tests for each individual. Detailed qPCR results for all six individuals (Individual 1–6). Standard deviation (SD) of cycle thresholds (Cq) as calculated by BestKeeper. geNorm results in M values (suggested upper threshold indicated by dashed line) as calculated by the algorithm. NormFinder results in Stability value as calculated by the algorithm.
(EPS)

S3 Fig qPCR results of reference gene tests for foot and head tissue. Detailed qPCR results for Foot tissue set and Head tissue set. Standard deviation (SD) of cycle thresholds (Cq) as calculated by BestKeeper. geNorm results in M values (suggested upper threshold indicated by dashed line) as calculated by the algorithm. NormFinder results in Stability value as calculated by the algorithm. First column represents spring animals (Individuals 1–3), second column represents autumn animals (Individuals 4–6), third column represents all individuals (Individuals 1–6).
(EPS)

S4 Fig qPCR results of reference gene tests for pigmented and unpigmented mantle tissue. Detailed qPCR results for Pigmented Mantle tissue set and Unpigmented Mantle tissue set. Standard deviation (SD) of cycle thresholds (Cq) as calculated by BestKeeper. geNorm results in M values (suggested upper threshold indicated by dashed line) as calculated by the algorithm. NormFinder results in Stability value as calculated by the algorithm. First column represents spring animals (Individuals 1–3), second column represents autumn animals (Individuals 4–6), third column represents all individuals (Individuals 1–6).
(EPS)

S5 Fig qPCR results of reference gene tests for body (F + H) and mantle (PM + UPM) tissue. Detailed qPCR results for Body tissue set and Mantle tissue set. Standard deviation (SD) of cycle thresholds (Cq) as calculated by BestKeeper. geNorm results in M values (suggested upper threshold indicated by dashed line) as calculated by the algorithm. NormFinder results in Stability value as calculated by the algorithm. First column represents spring animals (Individuals 1–3), second column represents autumn animals (Individuals 4–6), third column represents all individuals (Individuals 1–6).
(EPS)

S6 Fig qPCR results of reference gene tests for mantle and foot (PM + UPM + F) and mantle and head (PM + UPM + H) tissue. Detailed qPCR results for Mantle and Foot tissue set and Mantle and Head tissue set. Standard deviation (SD) of cycle thresholds (Cq) as calculated by BestKeeper. geNorm results in M values (suggested upper threshold indicated by dashed line) as calculated by the algorithm. NormFinder results in Stability value as calculated by the algorithm. First column represents spring animals (Individuals 1–3), second column represents autumn animals (Individuals 4–6), third column represents all individuals (Individuals 1–6).
(EPS)

S7 Fig qPCR results of reference gene tests for all tissues (PM + UPM + F + H). Detailed qPCR results for all tissue types combined. Standard deviation (SD) of cycle thresholds (Cq) as calculated by BestKeeper. geNorm results in M values (suggested upper threshold indicated by dashed line) as calculated by the algorithm. NormFinder results in Stability value as calculated by the algorithm. First column represents spring animals (Individuals 1–3), second column represents autumn animals (Individuals 4–6), third column represents all individuals (Individuals 1–6).
(EPS)
