# Supplementary figures and images for: Identification and validation of reference genes for qPCR in the terrestrial gastropod Cepaea nemoralis
Source: PLoS One. 2018 Aug 29;13(8):e0201396. doi: 10.1371/journal.pone.0201396 (PMC6114279; doi:10.1371/journal.pone.0201396)

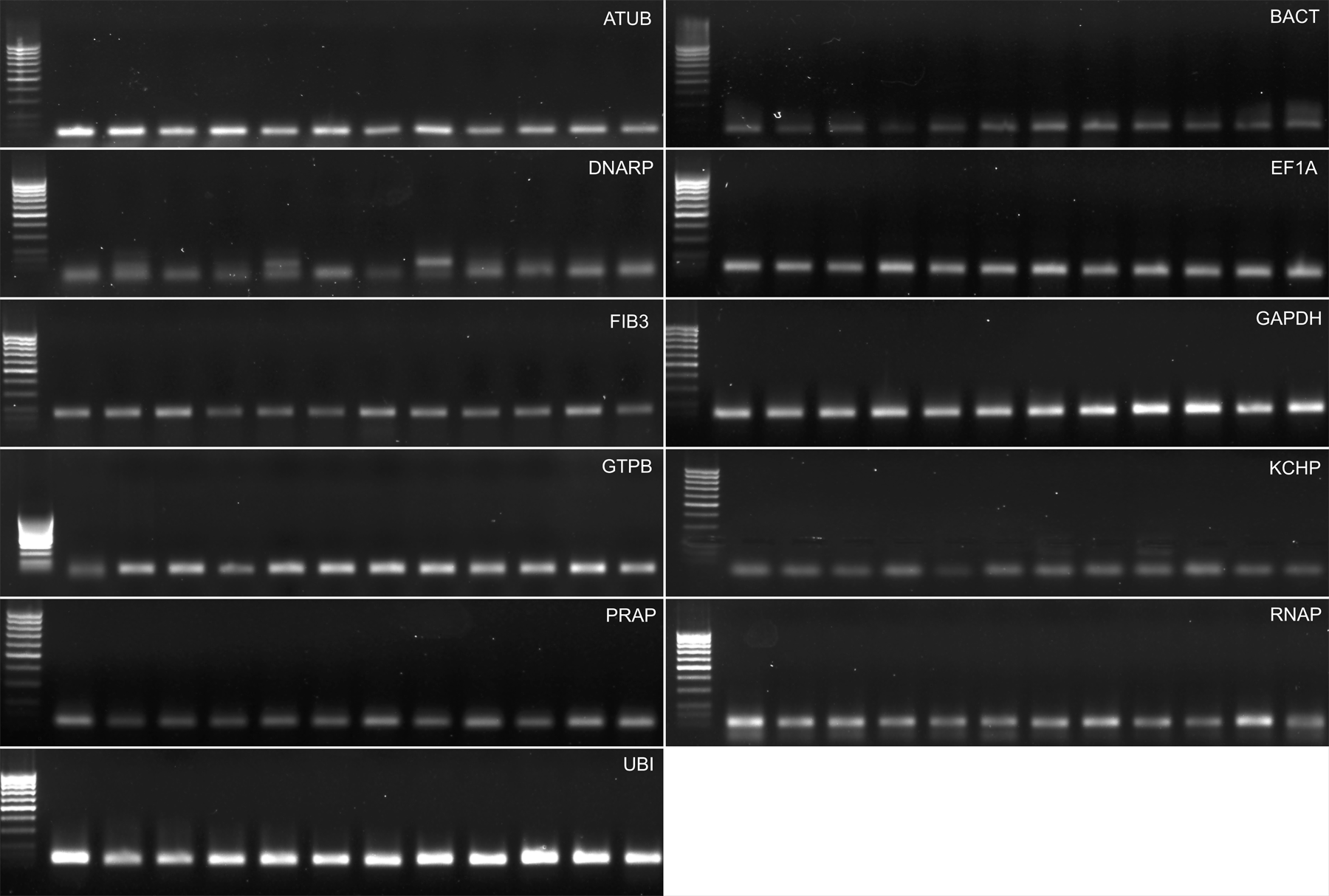

Supplement: S1 Fig — Representative images of agarose gel electrophoresis after qPCR amplification for each of the tested reference genes. Size ladders on the left indicate fragments from 100 to 1000 base pairs. (TIF) [file pone.0201396.s002.tif]
